# Beyond PRNU: Learning Robust Device-Specific Fingerprint for Source Camera Identification

**DOI:** 10.3390/s22207871

**Published:** 2022-10-17

**Authors:** Manisha, Chang-Tsun Li, Xufeng Lin, Karunakar A. Kotegar

**Affiliations:** 1 Department of Data Science and Computer Applications, Manipal Institute of Technology, Manipal Academy of Higher Education, Manipal 576104, India; 2 School of Information Technology, Deakin University, Geelong 3216, Australia

**Keywords:** image forensics, source-camera identification, PRNU, deep learning, convolutional neural network

## Abstract

Source-camera identification tools assist image forensics investigators to associate an image with a camera. The Photo Response Non-Uniformity (PRNU) noise pattern caused by sensor imperfections has been proven to be an effective way to identify the source camera. However, the PRNU is susceptible to camera settings, scene details, image processing operations (e.g., simple low-pass filtering or JPEG compression), and counter-forensic attacks. A forensic investigator unaware of malicious counter-forensic attacks or incidental image manipulation is at risk of being misled. The spatial synchronization requirement during the matching of two PRNUs also represents a major limitation of the PRNU. To address the PRNU’s fragility issue, in recent years, deep learning-based data-driven approaches have been developed to identify source-camera models. However, the source information learned by existing deep learning models is not able to distinguish individual cameras of the same model. In light of the vulnerabilities of the PRNU fingerprint and data-driven techniques, in this paper, we bring to light the existence of a new robust data-driven device-specific fingerprint in digital images that is capable of identifying individual cameras of the same model in practical forensic scenarios. We discover that the new device fingerprint is location-independent, stochastic, and globally available, which resolves the spatial synchronization issue. Unlike the PRNU, which resides in the high-frequency band, the new device fingerprint is extracted from the low- and mid-frequency bands, which resolves the fragility issue that the PRNU is unable to contend with. Our experiments on various datasets also demonstrate that the new fingerprint is highly resilient to image manipulations such as rotation, gamma correction, and aggressive JPEG compression.

## 1. Introduction

The availability of cost-effective smartphones has made the creation of digital images easier than ever before. As a result, digital images have become ubiquitous. Sometimes, images are used for malicious purposes such as fake news creation, pedo-pornography, and violence instigation. In such cases, investigators may be interested in identifying the camera that was used to capture the image in question. However, one can easily manipulate the image using freely accessible photo editing tools without leaving any visually detectable traces. This reduces the credibility of digital images to serve as valid evidence in a court of law. In such a crime-scene investigation, source camera identification tools assist the forensic investigator to ensure the trustworthiness of the digital image in question by identifying the origin of the image. Despite the fact that information about the camera model, date, and time are available in the image header file (i.e., the metadata or EXIF data), this cannot be used for forensic purposes, as it can be easily tampered with. As a result, blind approaches have been developed by investigating self-contained image data to identify the source camera rather than relying on auxiliary metadata [1]. Blind-source identification techniques take advantage of the subtle traces left on the image by various modules involved in the image acquisition pipeline. These traces carry certain information that is unique to the source camera of the image and can hence be used as a device fingerprint.

Different camera models employ different lens systems to focus the light on the sensor, resulting in lens distortion in the image [2]. Each camera model has a unique lens distortion pattern that helps identify the camera model for a given image. Since the camera sensor can measure only one color at each pixel location, a Color Filter Array (CFA) is used so that the individual pixels only receive the light of a certain wavelength. The missing color information is estimated from the neighboring pixels through a process called demosaicing (interpolation). Camera models from different brands have their own demosaicing algorithms to reconstruct the missing color values. Thus, inter-pixel dependencies caused by CFA demosaicing have also been used to identify the camera model of an image [3,4,5,6]. Cameras from different brands follow proprietary post-processing algorithms such as white balancing and JPEG compression. Hence, the statistical traces left on image by post-processing operations have been used to identify the camera model of the image as well [7,8,9].

However, the aforementioned intrinsic traces can only differentiate the cameras of different brands or models and cannot trace back to individual devices belonging to the same brand and model. Thus, it is necessary to extract features that can identify the exact camera. Imperfections in camera sensor manufacturing lead to variation in different pixels’ sensitivity to light of the same intensity. As a result, each camera leaves unique Sensor Pattern Noise (SPN) [10] in every image that it captures. The SPN has two major components: Fixed Pattern Noise (FPN) and Photo Response Non-Uniformity (PRNU). The FPN and PRNU depend on dark current and non-uniformity in the pixels of the camera sensor, respectively. The FPN caused by the dark current in the camera sensor is usually suppressed using a dark frame within the camera. Therefore, PRNU is the only dominant component of the SPN that is present in the output image. The PRNU noise pattern, being a deterministic component, is stable over time and remains approximately the same if multiple images of the same scene are captured [10]. It is independent of scene detail in the image and depends only on the physical characteristics of the camera sensor. Thus, each camera is characterized by its unique PRNU, which can be used as a device fingerprint for differentiating individual cameras of the same model or brand [10]. Due to these distinct characteristics, the PRNU has drawn much attention in device-level source-camera identification.

Over the past two decades, a variety of methods have been proposed to identify source cameras by extracting the PRNU from images [10,11,12,13]. Since the PRNU resides in the high-frequency band of the image, most source-identification techniques [12,14,15,16] employ the denoising filter proposed in [10] to extract the PRNU from an image. In [13], Li and Li further improved source-identification accuracy by preventing interpolation noise from contaminating the PRNU fingerprint. The PRNU, a unique camera fingerprint, has been used for many other forensic purposes such as detection of forgeries, face recognition [17], video source attribution [18], and clustering images uploaded by users on social networks [19]. Using the PRNUs from each color channel, Hou and Lee [20] proposed a forensic strategy for the detection of hue modification in images. Iuliani et al. [21] used the PRNU fingerprint generated from still images to identify the video source. Pande et al. [22] developed a hardware architecture to perform video source identification using PRNU noise and demonstrated acceleration of the task, making it suitable for real-time application. However, the PRNU, a form of hand-crafted device fingerprint, can be attenuated by many factors, such as scene details [23], PRNU filtering [14], periodic image processing operations [15], camera settings [24], etc., and can be easily removed to hinder source identification through simple low-pass filtering [25,26]. Moreover, the extracted PRNU pattern has the same pixel number as the original image, which incurs high computational and storage costs for source-oriented image clustering [27,28,29]. To overcome the computational complexity, the PRNU is either extracted from a small portion of the image or is formatted as a more compact representation [27,30]. However, this results in the loss of important features characterizing the source camera, thus compromising the accuracy of source-camera identification. Further, Al Shaya et al. [31] showed the difficulties in identifying the source of High Dynamic Range (HDR) images by using PRNU-based techniques.

Although the PRNU fingerprint has been proven to be an effective way to identify a source camera, it may be undesirable for anti-forensic or criminal attackers such as pedophiles or fake news creators who want to retain their anonymity when sharing images. It would be desirable to unlink images from their source camera when anti-forensics is needed. Thus, various counter-forensic techniques have been developed that eliminate or suppress the PRNU to anonymize images. Further, desynchronization operations such as rotation, cropping, resizing, and filtering prevent the detection of a PRNU fingerprint by disturbing the relationship between the neighboring pixel, thereby hindering correct identification of the source camera. There is currently a range of approaches in the literature [25,26,32,33] that are effective in defeating state-of-the-field source-camera identification techniques by eliminating the PRNU fingerprint. A forensic investigator having no idea of a counter-forensic attack on the image would have a high probability of being misled by the attacker.

In recent years, inspired by the success of deep learning in computer vision, image forensics researchers developed Convolutional Neural Network (CNN)-based data-driven systems [34,35,36,37,38,39,40] that can automatically extract features to identify the source. Nevertheless, CNNs are susceptible to adversarial attacks, which also calls into question the reliability of existing forensic methods. For example, Xie et al. [41] adopted a Generative Adversarial Nets-based anti-forensic framework that aims to erase operation-specific traces in order to improve the undetectability of generated images. Wang et al. [42] proposed a powerful adversarial attack to deceive the source-camera identification task. Furthermore, current state-of-the-art CNN-based approaches [43,44,45,46,47] provide promising solutions to camera model identification. However, as we discuss in Section 2, they are not capable of effectively identifying devices of the same model or brand. Therefore, it is desirable to have a robust fingerprint that is capable of differentiating individual devices of the same model. The major contributions of the proposed work are as follows.

1. We make evident the presence of a new *non-PRNU* device-specific fingerprint in digital images and develop a data-driven approach using CNN to extract the fingerprint.
2. We show that the new device-specific fingerprint is mainly embedded in the low- and mid-frequency components of the image; hence, it is more robust than a fingerprint (e.g., PRNU) residing in the high-frequency band.
3. We show that the *global, stochastic, and location-independent* characteristics of the new device fingerprint make it a robust fingerprint for forensic applications.
4. We also demonstrate the reliability of the new device-specific fingerprint in real-world forensic scenarios through open-set evaluation.
5. We validate the robustness of the new fingerprint on common image processing operations.

The remainder of the paper is structured as follows. Section 2 reviews related works. Section 3 presents the proposed data-driven approach to explore the presence of a new fingerprint. Experimental results and discussions are given in Section 4. Finally, conclusions are drawn in Section 5.

## 2. Literature Review

In light of the high sensitivity of PRNU (i.e., a form of hand-crafted device fingerprint) to common image processing (e.g., low-pass filtering and compression) in source-camera identification, the past few years have witnessed successful attempts at taking data-driven approaches based on CNN [34,35,36,37,38,39]. Bondi et al. [34] proposed a model based on CNN and SVM for camera model identification. The method aimed at learning camera model-specific features directly from the image rather than depending on hand-crafted features. The proposed method was able to work on small image patches (64 × 64 pixels) with 93% accuracy on 18 different camera models. Tuama et al. [35] developed a CNN model by modifying AlexNet. A pre-processing layer consisting of a high-pass filter was added to the CNN model to reduce the impact of scene details. The CNN was trained on image patches of size 256 × 256 pixels to identify the model of the source camera. A CNN-based robust multi-classifier was developed by Yao et al. [36] to identify the camera model. The method used 256 non-overlapping patches of size 64 × 64 pixels extracted from the central portion of the image and achieved a classification accuracy of nearly 100% over 25 camera models. Freire-Obregón et al. [37] proposed a source-camera identification method for mobile devices based on deep learning. The method involved training the CNN model using 256 image patches of size 32 × 32 pixels extracted from each image. The performance was degraded when multiple cameras of the same brand and model were considered. Huang et al. [38] developed a CNN model and evaluated the effect of the number of convolutional layers on the performance of the model. The work suggested that the deeper CNN can achieve better classification accuracy. Further, the work showed that replacing the softmax layer in the CNN with the SVM classifier improved identification accuracy. Wang et al. [39] developed a CNN model by modifying AlexNet and equipping it with a Local Binary Pattern (LBP) pre-processing layer to allow the CNN to have more focus on the intrinsic source information (such as PRNU [10], lens distortion noise pattern [2], and traces of color dependencies related to CFA interpolation [3]) that is concealed in the image rather than the scene details. The images were divided into non-overlapping patches of size 256 × 256 pixels to train the CNN model. The proposed method achieved an identification accuracy of 98.78% over 12 camera models. Liu et al. [48] proposed a representative patch selection method based on multiple criteria to reduce the training costs involved in deep learning-based source identification. Albeit encouraging that the aforementioned CNN-based methods are able to effectively identify the model of the source camera of the images in question, they are by no means capable of effectively differentiating individual cameras of the same model.

In [43], Chen et al. proposed a method to investigate the task of brand, model, and device identification using a residual network. The model was trained using one patch of size 256 × 256 extracted from each image. Despite having good ability to identify the brand and model, it was able to achieve only 45.81% accuracy when different devices of the same brand and model were involved in the experiment. Yang et al. [44] developed a content-adaptive fusion network to identify source devices of small image patches of size 64 × 64. The exact device detection accuracy was 70.19% when only three devices of the same model were used. Ding et al. [45] further improved the task of exact device identification by developing a residual network with a multi-task learning strategy by taking advantage of both hand-crafted and data-driven technologies. However, the model was able to achieve only 52.4% accuracy on 74 cameras from the Dresden dataset and 84.3% accuracy on 51 cameras from the Cellphone dataset on image patches of size 48 × 48 pixels.

In almost all the existing techniques, a large number of images are used to train the CNN models for source identification. However, collecting a large number of images is infeasible in realistic scenarios. Thus, Sameer et al. [46] addressed the task of source-camera identification with a limited set of images per camera. A deep Siamese network was trained using a few-shot learning technique by considering pairs of 64 × 64 pixel image patches extracted from 10 images per camera. The method outperformed other state-of-the-art techniques with few training samples. Mandelli et al. [47] proposed a two-channel-based CNN that learns a way of comparing image noise residual and the camera fingerprint at the patch level. Further, the method considered the scenario in which PRNU fingerprint and images are geometrically synchronized. The method was effective in achieving enhanced source identification accuracy. However, it depends on the PRNU fingerprint for source camera identification. Hence, it works only when there is no pixel misalignment between the PRNU fingerprint and when the PRNU has not been attacked.

Although much progress in differentiating individual devices [43,44,45,46,47] using the data-driven approach has been made to address PRNU’s fragility issue, the accuracy is still far from forensically satisfactory. Thus, extracting the features/fingerprints for individual device identification remains a challenging task. Furthermore, existing CNN models for source identification work only when no pixel misalignment is present in the images. However, image manipulations and counter-forensic attacks can defeat state-of-the-art source-camera identification techniques by breaking the structural relationship between neighboring pixels and eliminating the PRNU noise embedded in the image. Moreover, such a spatial synchronization requirement (i.e., all image patches need to be taken from the same location) is in no way feasible if the original image has been subjected to cropping or affine transformations such as scaling, rotation, shearing, translation, etc. In view of the vulnerabilities of PRNU to counter-forensic attacks and common image processing techniques, the research question then arises: **is there some form of robust device-specific fingerprint other than PRNU in an image that is capable of differentiating individual devices of the same model?** To answer this question, we propose two simple, yet effective, methods, *downsampling* and *random sampling*, to generate PRNU-free images for learning robust device-specific fingerprints. By leveraging the generated PRNU-free images and the powerful learning capability of deep residual neural networks, we can establish the presence of robust device-specific fingerprints other than PRNU that can be used to identify the exact source device of images.

## 3. Methodology

As we aim for the proposed model to learn a robust device fingerprint, our approach is to avoid the high-frequency bands of images. This is also to serve the purposes of (1) proving that the new device fingerprint is PRNU-irrelevant (i.e., its effectiveness is not attributed to the PRNU) and (2) enhancing the new device fingerprint’s immunity to the interference of strong scene details appearing in the high-frequency bands. In addition to this, every day a large number of images are shared among users through social networks such as Facebook, WhatsApp, and Instagram. Each social network has its proprietary compression standards that are applied while uploading and downloading images. The JPEG and proprietary compression standards widely used on social networks significantly affect the high-frequency band of images. This poses new challenges in source identification tasks, as these actions tend to damage the PRNU fingerprint residing in the high-frequency band. Therefore, we present two different ways of creating PRNU-free image patches in Section 3.1 and Section 3.2 to reveal different characteristics of the new device fingerprint and to align patch formation methods naturally with the input requirement of the proposed deep learning model (i.e., Section 3.3). The two ideas of forming PRNU-free image patches are applicable to other learning models.

### 3.1. Generation of PRNU-Free Images with Down Sampling

**Figure 1:**
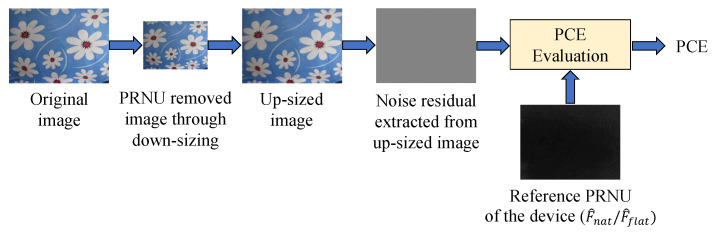
PRNU fingerprint removal through downsampling and removal effectiveness evaluation based on PCE.

The PRNU fingerprint is embedded in the high-frequency bands of images [10]. Hence, removal of the PRNU fingerprint can be achieved by eliminating the high-frequency components of the image. There are various filtering operations for removing high-frequency components of images. Since we use ResNet as the fingerprint extractor (see Section 3.3), which require patches of 224 × 224 pixels as input, our first method for PRNU removal is downsampling (a form of low-pass filtering), which serves not only to remove the PRNU but also conforms to the ResNet architectural requirement. We apply bilinear filtering (low-pass filtering) to downsample each image to 224 × 224 pixels with three color channels. To ensure effective PRNU removal, we evaluate the similarity between the noise residual extracted from the PRNU-free image and the reference PRNU of its source camera using the widely adopted Peak-to-Correlation Energy (PCE) [11], as depicted in Figure 1. The experiments in [11] suggested a PCE detection threshold of 50 to determine whether two images are from the same source device. The greater the PCE value is, the higher the likelihood that the two images are of the same origin. Further, counter-forensic attacks used to impede PRNU often fix the PCE detection threshold at 50 to ensure effective PRNU removal, thereby unlinking the images from their source cameras [25,32]. Thus, in the proposed work, we also use 50 as the threshold to determine whether the downsampled images have become PRNU-free.

PCE evaluation requires the noise residual that serves as the PRNU fingerprint of the image ($I_{D}$) and the reference PRNU fingerprint of its source device (*D*). We use wavelet-based denoising [11] for noise-residual extraction and construct the reference PRNU (${\hat{F}}_{nat}$) from the noise residuals extracted from 50 natural images captured by the same device. To calculate the PCE value, we upsample the downsampled PRNU-free images to the same size as the reference PRNU. We also consider the reference PRNU (${\hat{F}}_{flat}$) generated using 50 flat-field images. Flat-field images have fewer intensity variations, which results in the generation of near-perfect camera fingerprint, making the PCE estimate more convincing. As we explain in Section 4.2, the downsampling operation results in PCE values much lower than the threshold of 50, indicating the effectiveness of PRNU removal in the downsampled images.

### 3.2. Generation of PRNU-Free Images with Random Sampling

Patch formation based on downsampling can help our search for device fingerprints in the low- and mid-bands of images. However, it preserves the spatial relationship among the pixels within the patches and is not able to address the spatial synchronization issue that the PRNU is unable to contend with. Therefore, in this second method, we propose using random sampling to remove the PRNU fingerprint in images while complying with the ResNet architectural requirement. More importantly, we intend to prove that there is a form of device fingerprint that is location-independent, stochastic, and globally present. Being a location-dependent and deterministic component [10,49], the PRNU fingerprint requires proper spatial synchronization; thus, forming image patches with pixels selected at random causes desynchronization and hence prevents the proposed residual network from learning the PRNU fingerprint. Furthermore, the random sampling operation disrupts the contextual and semantic information of the image, which is irrelevant to the intrinsic fingerprint of the source device. By so doing, the model learns the device-specific fingerprint while ignoring irrelevant but interfering information. For this purpose, we create patches of size 224 × 224 × 3 pixels by taking pixels from random locations either from the original image $I_{D}$ or the downsampled image upsampled to original size ${\hat{I}}_{D}$, thereby eliminating the structural relationship between neighboring pixels. Here, each pixel is used only once while forming the patches. If a machine learning model is able to learn from the image patches formed by taking the pixels at random for device-level source camera identification, we make evident the global presence of location-independent, stochastic, and PRNU-irrelevant device-specific fingerprint that can be successfully used for image forensic applications.

### 3.3. Learning Device-Specific Fingerprints

With the PRNU-free images generated using the aforementioned two methods, we propose to use a hybrid system based on a residual neural network (ResNet) [50] and SVM classifier. ResNet is a variant of the CNN model with “shortcut/skip connections” that allows construction of substantially deeper networks for performance gain and has achieved state-of-the-art results on a range of computer vision benchmarks. The ResNet model serves as a fingerprint extractor to extract device-specific fingerprints that are then used to train an SVM classifier to verify the effectiveness of the new fingerprint in identifying the source camera. The hybrid CNN-SVM model makes up for the limitations of the CNN and SVM classifiers by integrating the merits of both classifiers. The learning algorithm of CNN tries to minimize the error on the training set. When the back-propagation algorithm finds the first decision boundary, regardless of whether it is the global or a local minima, the model discontinues the learning process and does not proceed to improve the decision boundary. Thus, the generalization capability of CNN is lower than that of SVM, as SVM tries to reduce the generalization errors on the unseen data. It finds a separating hyperplane by maximizing the margin between two classes of the training set. In this work, we develop a hybrid ResNet–SVM model to bring out their best abilities for source-camera device identification. Further, we empirically find that employing ResNet101 and SVM together increases classification accuracy by an average of 3 percentage points compared to using ResNet101 alone to identify sources.

The depth of the CNN is a crucial parameter [36,38] that significantly affects the performance of the source-camera identification task. However, building a deeper CNN is not as simple as stacking layers in light of the vanishing-gradient problem for neural networks relying on gradient-based training, e.g., back propagation. More specifically, greater network depth makes it increasingly difficult to propagate the gradients back to earlier layers of the network for weight updates. As a consequence, the performance of the CNN saturates or even starts degrading rapidly as the CNN network goes deeper. Such an issue can be addressed by introducing skip connections between layers [50], which add the outputs of earlier layers to the deeper layers and thus allow uninterrupted gradient flow between layers in the network. This not only tackles the vanishing-gradient problem but also enables the reusability of features at different layers [51], making it possible to learn both low-level and high-level features. Thus, our strategy is to make use of the deeper ResNet model to learn richer feature representation for extracting the device-specific fingerprint.

**Figure 2:**
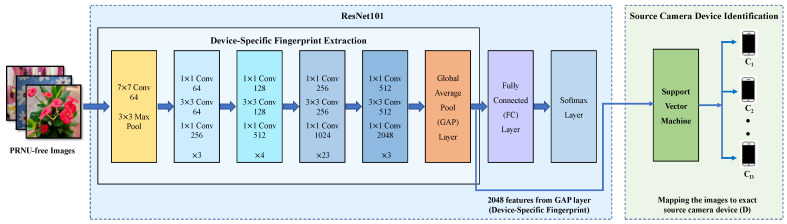
The hybrid ResNet–SVM to extract the new device-specific fingerprint without using PRNU to identify the source camera.

Feature/fingerprint extraction plays a major role in the success of source camera identification. It necessitates that extracted features exhibit discriminative characteristics among different cameras while retaining a high degree of homogeneity within the same class. The discriminative power of the features/fingerprints can be boosted by increasing the number of stacked layers. ResNet has many variants with different numbers of layers such as ResNet18, ResNet34, ResNet50, ResNet101, etc. With an increase in the number of layers, ResNet can integrate multiscale features from multiple layers to enhance feature discriminative power. In this work, we employ the ResNet model with 101 layers (ResNet101) as the backbone of our device-fingerprint extractor. The proposed ResNet-based device-specific fingerprint extractor is shown on the left side of Figure 2.

In order to capture the device-specific fingerprint without using the PRNU, we train ResNet101 using PRNU-free images as described in Section 3.1 and Section 3.2. The number of neurons in the last fully connected (FC) layer is equal to the number of devices *D* used for training the model. During forward propagation, the PRNU-free images are passed through successive ResNet blocks and the Global Average Pool (GAP) layer. Eventually, at the FC layer, the feature vector is reduced to the number of cameras *D* to get $z=[z_{1},z_{2},\ldots z_{D}]$. Finally, a softmax function is applied to convert the scores produced by the FC layer to probabilities $\left(\hat{y}=\left[{\hat{y}}_{1},{\hat{y}}_{2},\ldots {\hat{y}}_{D}\right]\right)$, where ${\hat{y}}_{i}$ is the probability of the image being taken with the *i*th camera:

(1) $${\hat{y}}_{i}=\frac{e^{z_{i}}}{\sum_{d=1}^{D}e^{z_{d}}}$$

The prediction loss is then calculated based on the class distribution ($\hat{y}$) predicted by ResNet101 and the one-hot encoded ground-truth class vector (*y*) of the input image. For the proposed work, we make use of the *categorical cross-entropy loss function*, which is widely used for multi-class classification:

(2) $$L\left(\hat{y},y\right)=-\sum_{i=1}^{D}y_{i}\log\left({\hat{y}}_{i}\right)$$

where $y_{i}$ and ${\hat{y}}_{i}$ are the actual class and the probability of the input image belonging to the *i*th camera, respectively. In the proposed work, we extract the feature vector from the GAP layer of ResNet101 to serve as the device-specific fingerprint. This results in the extraction of a 1 × 1 × 2048 dimensional feature map from each image. Here, 2048 indicates the number of output channels (number of filter/kernels) of the last convolution (Conv) layer of ResNet101, which remains fixed for any input image. The last convolution layer in ResNet101 uses 2048 filters of size 1 × 1. The GAP layer reduces the dimension of the feature map by taking the average of each feature map produced by the previous layer. Thus, the dimension of the feature vector remains fixed every training time. Therefore, a 2048-dimensional feature vector extracted from the GAP layer serves as the robust device-specific fingerprint, which is further used to identify the exact source device of images using the SVM classifier, as we discuss in Section 3.4.

### 3.4. Verification of Fingerprint’s Effectiveness in Source-Camera Identification

To make it evident that the device fingerprint extracted by ResNet101 can identify individual devices, we use a multi-class SVM classifier with a Radial Basis Function (RBF) kernel to identify the source device of PRNU-free images. We feed the images to the trained ResNet101 model and use the outputs from the GAP layer as the device-level fingerprints for training the SVM classifier. Once the SVM classifier is trained, it makes class predictions on the images in the test set based on the device fingerprint extracted from the GAP layer. We use the same training set (75% of the images of each camera) for training both ResNet101 and the SVM classifier, while the testing set (25% of the images of each camera) is only used for testing the effectiveness of the fingerprint extractor and source-camera identification performance of the SVM classifier, which ensures that the testing set is not involved in the training of the fingerprint extractor (i.e., ResNet101).

## 4. Experiments and Discussions

### 4.1. Experimental Setup

For evaluation of the proposed method, we used images from the VISION dataset [52], Warwick Image Forensic dataset [53], Daxing dataset [54], UNISA2020 [55], and a custom-built dataset. All datasets have different characteristics in terms of camera model and brand, image content, and number of devices of the same model. Because our work aims at identifying device-specific fingerprints, we considered only images taken from different devices belonging to the same brand and model in these datasets. Details of the cameras and the images used are given in Table 1. From the VISION dataset, in addition to natural images, we also considered 50 flat-field images for generation of the reference PRNU (${\hat{F}}_{flat}$). The custom-built dataset includes images taken from two personal smartphone models. From each model, we considered two different devices. Involving different datasets in the experiments ensures the diversity of the cameras and images used for evaluation. For each dataset, the images are randomly divided into training and testing sets, such that 75% of the images from each device are randomly chosen for training the model, and the remaining 25% are for testing the model. We train ResNet101 for 20 epochs using the Adam optimizer with the mini-batch size and the learning rate set to 12 and 0.001, respectively. All experiments are conducted using Matlab2020b on an HP EliteDesk 800 G4 Workstation with an NVIDIA GeForce GTX 1080 GPU, 3.7 GHz Intel processor, and 32 GB RAM.

### 4.2. Evaluation of Downsampled Patches

For each camera, the reference PRNU (${\hat{F}}_{nat}$) utilized during the PCE evaluation is estimated using 50 natural images. For the VISION dataset, we also generate the reference PRNU (${\hat{F}}_{flat}$) using 50 flat-field images. To evaluate the effectiveness of PRNU removal through image downsampling, we compare the average PCE values over 30 test images before and after removing the PRNU with the PRNU removal method proposed in Section 3.1. From the results in Table 2, we can see that there is a substantial reduction in the PCE values after removing the PRNU from the images. Be it the reference PRNU ${\hat{F}}_{nat}$ estimated from natural images or ${\hat{F}}_{flat}$ estimated from flat-field images, the average PCE values for the downsampled images are significantly less than the detection threshold of 50, indicating the effectiveness of the proposed downsampling PRNU removal method.

**Figure 3:**
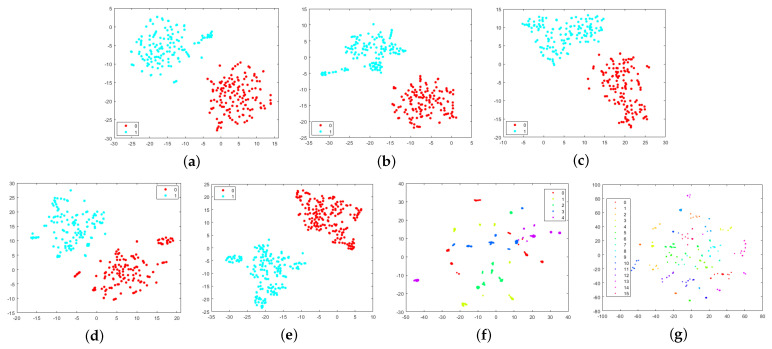
Results obtained with t-SNE on different datasets demonstrating the separation between different devices belonging to the same model: (**a**) two iPhone 5c devices, (**b**) two iPhone 4s devices, (**c**) two Fujifilm X-A10 devices, (**d**) two Redmi Note 3 devices, (**e**) two Asus Zenfone Max Pro M1 devices, (**f**) five Xioami 4A devices, and (**g**) 16 devices of 6 different models from the Daxing dataset.

We further present our results on the establishment of a new non-PRNU device-specific fingerprint. We have shown that downsampling is effective in removing PRNUs. Thus, to demonstrate that device-specific fingerprints can be effectively extracted from PRNU-free images, we first downsample all images in the training and testing sets to 224 × 224 × 3 pixels and then train ResNet101 on these PRNU-free images from different devices of the same camera model. We carry out various experiments by training ResNet101 on different datasets. The devices used for each experiment are listed in Table 3. The feature extracted from the GAP layer of the trained ResNet101 model is 2048-dimensional. To visualize high-dimensional features, we use t-distributed Stochastic Neighbor Embedding (t-SNE) and show the results in Figure 3. As we can see, the separation between the devices belonging to the same brand and model is well evidenced, which suggests that *there is a unique device-specific fingerprint other than PRNU in the images*.

Furthermore, the downsampling operation removes the high-frequency components in the images, so only the low- and mid-frequency components of the images were taken into account by ResNet101 while learning the non-PRNU device fingerprint. This indicates that the features that serve as *the device-specific fingerprint extracted by the proposed system are mainly embedded in the low- and mid-frequency components of the image*. Since high-frequency components of the image are sensitive to manipulation and counter-forensic attacks, we cannot rely on device fingerprints concealed in the high-frequency band for forensic purposes. However, the proposed method makes evident the presence of a new device fingerprint in the low- and mid-frequency band that can be used as a robust fingerprint to identify the exact source device of images.

Similar to the work in [34], we train an SVM classifier on the 2048-dimensional features extracted by ResNet101 to test their capability in identifying the exact source device of images. The performance of the SVM classifier on exact device identification is evaluated in terms of classification accuracy and is reported in the third column of Table 3. It can be observed that the proposed model is able to map the PRNU-free images to their respective source cameras accurately based on the device-specific fingerprint extracted by ResNet101. Furthermore, the proposed model achieves the best testing accuracies of 99.57% and 99.20% for Experiment 6 and Experiment 7, respectively. Note that the 5 Xiaomi 4A devices in Experiment 6 and the 16 devices of 6 different models in Experiment 7 are from the Daxing dataset, which includes flat-field images such as the images of clear skies and white walls that are uniformly illuminated. As flat-field images have less intensity variation, the proposed deep learning model is less susceptible to scene details. This makes the model focus more on the intrinsic information of the images, which contributes to learning the fingerprint. As a result, the proposed model achieved higher accuracy on the Daxing dataset compared to the other datasets.

### 4.3. Evaluation on Randomly Sampled Patches

In this subsection, we evaluate the performance of the proposed system in extracting the new device-specific fingerprint from randomly sampled patches, as detailed in Section 3.2. To study whether or not there are global stochastic device-specific characteristics that can act as device fingerprints, from every image in the training and testing sets, we extract 50 patches with pixels taken at random from the original images or from downsampled images. The proposed hybrid ResNet–SVM system is trained on these randomly sampled patches to extract the non-PRNU device fingerprint and identify the source device. We repeat the same experiments as in the previous subsection for the proposed random-sampling method and report the results in the fourth and fifth columns of Table 3. Compared to the results of downsampling, the individual device identification accuracy of random sampling drops notably from about 95% to 85% for the cases with two devices. This is not surprising, as the local structural information of the original image is abandoned for training. However, what is most striking is that the identification accuracy (>83%) of random sampling is much higher than that of a random guess. Furthermore, the influence of the absence of structural information tends to diminish as the number of devices increases in Experiments 6 and 7.

**Figure 4:**
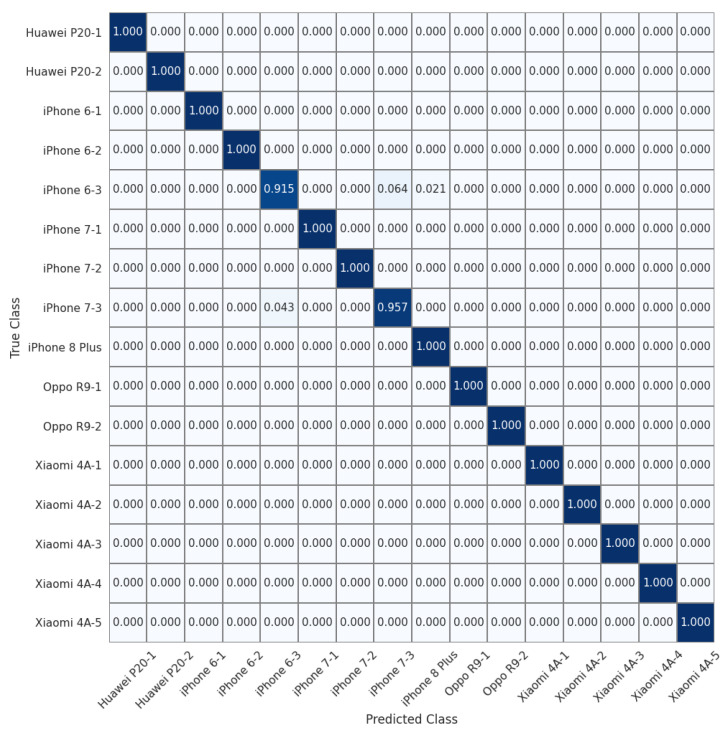
Normalized confusion matrix for source-camera identification based on downsampled images extracted from original images for 16 devices of 6 different models in the Daxing dataset.

**Figure 5:**
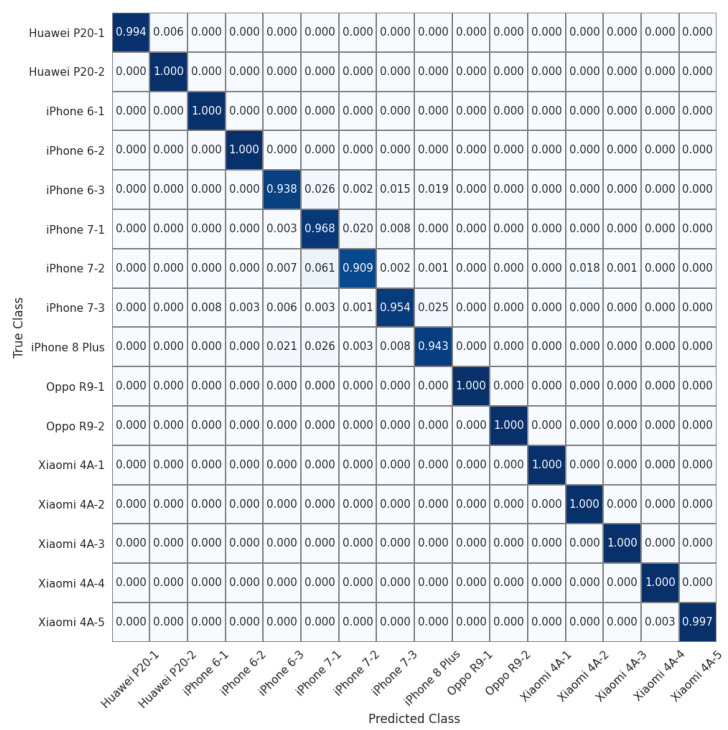
Normalized confusion matrix for source-camera identification based on randomly sampled images extracted from original images for 16 devices of 6 different models in the Daxing dataset.

**Figure 6:**
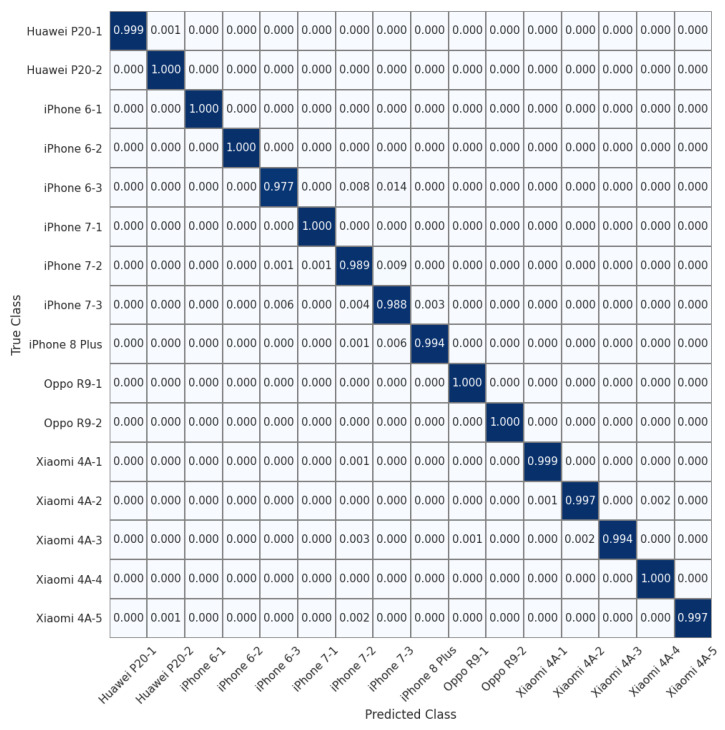
Normalized confusion matrix for source-camera identification based on randomly sampled patches extracted from downsampled images for 16 devices of 6 different models from the Daxing dataset.

To allow for a more detailed comparison, we show the normalized confusion matrices on the 16 devices of the Daxing dataset (Experiment 7) in Figure 4, Figure 5 and Figure 6. We can see that the source identification performance only drops slightly for a few iPhone devices, i.e., iPhone 7 and iPhone 8 Plus. These results appear to indicate that involving more devices in the training is beneficial for learning discriminative device-specific fingerprints. However, as we involve more devices in the training, we should not overlook the potential chance of learning model-specific rather than device-specific fingerprints, because the number of camera models is usually larger than the average number of devices of the same model in a dataset. Thus, further studies are required to investigate the appropriate way of constructing datasets for learning discriminative device-level fingerprints. Another interesting observation from Table 3 is that random sampling from the downsampled images slightly outperforms random sampling from the original images. We contend that most high-frequency components removed by downsampling can be considered interference for device-specific fingerprint extraction. Therefore, similar to the common practice of suppressing the influence of scene details on PRNU to improve source-camera identification [23], random sampling from the downsampled images rules out the effect of interferential high-frequency components in the images on the new device-specific fingerprint and thus improves device identification performance.

For random sampling, since each patch is formed with pixels taken at random from the image, the spatial relationship among the pixels within each of the patches is disturbed. Therefore, no structural information (e.g., model-specific periodic artifacts due to JPEG compression or demosaicing) is maintained in each patch. The results of random sampling in Table 3 suggest the existence of stochastic and location-independent device-specific characteristics that can be captured by random sampling to serve as device fingerprints. The PRNU fingerprint, being a deterministic component, requires proper spatial synchronization; post-processing, such as resizing, cropping, and filtering, of the images prevents authentic source identification based on PRNU. Thus, relying on the fragile PRNU for forensic purposes will mislead investigations. With the new characteristics being stochastic and globally present, it opens the opportunity to learn robust device-specific fingerprints that are less susceptible to desynchronization operations and counter-forensic attacks on images.

### 4.4. Open-Set Validation of New Device-Specific Fingerprint

One of the major drawbacks of existing source identification techniques is that they mainly cope with the closed-set scenario. This means that the image in question is always mapped to one of the cameras used to train the source identification system. However, in a practical scenario, the forensic analyst may be confronted with an image that was not taken with any of the cameras used during the training process. Thus, the forensic analyst must also be able to trace back the source camera in the open set that captured the image. In this vein, we present herein an in-depth analysis of the effectiveness of the new device-specific fingerprint in the open-set scenario. Specifically, we demonstrate the reliability of the new device fingerprint in handling the real-world forensic scenario of mapping the image in question to the suspect camera. To this end, we study the possibility of exploiting the proposed fingerprint extractor tailored to capture the new device-specific fingerprint as discussed in Section 4.2 to extract the features/fingerprint from images taken with a new set of cameras. Subsequently, we employ SVM as the open-set classifier to map images to their source cameras based on the features extracted by the proposed fingerprint extractor.

To evaluate the viability of the new device-specific fingerprint in the practical forensic scenario, we consider the fingerprint extractor trained on PRNU-free images (generated through downsampling) from (i) two devices of the iPhone 5c model and (ii) five devices of the Xiaomi 4A model. For the open-set evaluation, we consider the images taken with the cameras (as reported in Table 4) that are not used to train the fingerprint extractor and split them with a ratio of 75:25 to form the training and testing sets. We extract the features by a single pass through the downsampled images in the training set. By leveraging the 2048-dimensional device fingerprints extracted from the training images by the fingerprint extractor, we train the open-set classifier to associate the fingerprints to the respective source cameras. Once the classifier is trained on the features extracted from the images captured with the cameras in the open-set, it can be employed to map the images in the test set to their source cameras. In Table 4, we report the testing accuracy achieved in the open-set scenario by exploiting the proposed fingerprint extractor. The results clearly demonstrate the effectiveness of the new device-specific fingerprint in identifying the source camera in the open-set scenario. We can also observe that the identification accuracy tends to decrease as the number of devices increases. However, in a practical forensic investigation, the number of suspect cameras belonging to the same model is usually limited. What is astonishing about the results in Table 4 is that, even trained on a small number of cameras, the fingerprint extractor is able to extract device-specific fingerprints that are capable of distinguishing different devices of the same model with high accuracy (>90%) in an open-set scenario. Therefore, the experimental results demonstrate that the proposed fingerprint extractor does not overfit to the training data and is able to extract non-PRNU fingerprints that are effective for device-level source-camera identification. This suggests that the information ResNet101 works on is indeed device-specific and does not depend on dataset artifacts such as image resolution and/or scene details.

### 4.5. Analysis of Effect of PRNU Removal on State-of-the-Art Techniques

The existing literature on source-camera identification [34,35,36,37,38,39,43,44,45,46,47] has shown excellent performance on camera model identification. However, our preliminary analysis showed that the state-of-the-art techniques for camera model identification cannot be used directly to identify the exact device. Most, if not all, existing data-driven approaches [34,36,37,38,39,44] report a drastic reduction in device-level classification accuracy when PRNU-free images are used for training. In this subsection, we compare the performance of the proposed hybrid ResNet–SVM system in extracting the *non-PRNU* device fingerprint and identifying the source camera of PRNU-free images with the state-of-the-art data-driven approaches. We train the state-of-the-art techniques using PRNU-free images generated through the downsampling operation. The classification accuracy achieved on PRNU-free images in the testing set of different datasets is reported in Table 5. It can be observed that in the case of individual device identification, both the proposed downsampling and random sampling methods outperform the existing data-driven approaches by a large margin. We considered the deeper residual CNN with five convolutional blocks proposed in [43] specifically to perform exact device identification to investigate its feasibility in the absence of the PRNU fingerprint. Noticeably, on PRNU-free images, the approach shows low identification accuracy. The content-adaptive fusion network [44] developed for individual device identification is ineffective in differentiating different devices of the same model in the absence of the PRNU fingerprint. Further, the CNN proposed in [39] that learns intrinsic source information (such as PRNU, lens distortion noise pattern, and traces of color dependencies related to CFA interpolation) through the local binary pattern (LBP) preprocessing layer shows poor performance on PRNU-free images. Thus, their results indicate that the PRNU remains an important characteristic for the existing techniques to identify the source camera. They only work when a strong PRNU fingerprint is present in the image. Nonetheless, it can be seen that *our hybrid ResNet–SVM system is capable of identifying individual cameras of the same model from PRNU-free downsampled and randomly sampled image patches*.

Further, because the number of images from each camera in the UNISA2020 dataset is limited, we employ random sampling to generate PRNU-free images. Each camera in the UNISA2020 dataset has around 100 images that were captured at the highest available resolution (i.e., 4288 × 2848). We considered the images taken from four devices of the Nikon D90 model for the evaluation of the proposed system and state-of-the-art techniques. We create 50 patches from each image by taking pixels from random locations. The identification accuracy achieved on PRNU-free images generated through random sampling is reported in Table 6. It is interesting to see that the proposed system performed equally well on the aforementioned dataset. We also compared the performance with state-of-the-art methods using the same dataset. It can be observed that for individual device identification, the proposed system achieved 96.02% on four devices of the Nikon D90 model and outperformed the existing data-driven approaches by a large margin. Further, we evaluated the performance by considering ten devices of the Nikon D90 model from the same dataset and observed that the proposed system was still able to effectively identify the source camera with an identification accuracy of 92.96%. Thus, the best results achieved on the diverse range of datasets prove the stability of the proposed system in identifying the source camera.

We summarize two possible reasons that contribute to the superior performance of our proposed method: (1) Deeper networks allow learning device-level features that are capable of distinguishing different devices of the same brand and model. However, some existing works, e.g., [34,36,37,38,39,44], used shallow neural networks (less than 15 convolutional layers) to learn features, which might not be sufficiently discriminative for device-level source-camera identification. (2) Probably inspired by the success of PRNU, it is generally believed that intrinsic source device information largely resides in the noise residual or the high-frequency domain of an image. For this reason, most existing data-driven approaches [35,39,56,57,58] attempt to learn discriminative features from noise residuals or high-pass filtered images rather than from the original images. There is a chance that the CNN will learn strong scene details residing in the high-frequency band, which acts as interference. In contrast to these approaches, in the proposed work, ResNet101 learns from downsampled/randomly sampled images while ignoring such irrelevant but interfering information present in the high-frequency band. Therefore, the combination of the powerful learning capability of the deeper residual neural network and PRNU-free images contributes to the superior performance of our proposed method. Moreover, our proposed method successfully learns the device-specific fingerprint from PRNU-free images, which indicates that PRNU noise may only be one type, or make up a small part, of device-specific fingerprints.

### 4.6. Evaluation of Robustness against Image Manipulations

In practical circumstances, images are often subjected to various manipulations, which requires source identification techniques to be robust against these manipulations. In this subsection, we test the robustness of the proposed method against various image manipulations such as gamma correction (with γ∈{0.7,1.4}), rotation (with rotation angles $\theta \in \{15^{\circ },30^{\circ },90^{\circ }\}$), and JPEG compression (with quality factor Q∈{90%,50%,20%}) and thereby evaluating the capability of the new device fingerprint. To verify robustness, we consider the proposed system trained on the original downsampled images (i.e., no manipulations applied). For testing, all images in the test set are processed with the aforementioned image manipulations and then downsampled to remove PRNU fingerprints.

The effectiveness of the *non-PRNU* device-specific fingerprint in identifying the source camera of the manipulated images in terms of identification accuracy on different sets of cameras is reported in Table 7. It can be seen that the new fingerprint is effective in identifying images subjected to gamma correction. Identification accuracy of rotated images is slightly lower than that of the original images. JPEG compression is widely used on social networks, which suppresses the PRNU fingerprint left on the high-frequency band of the images, making source identification a difficult task. However, it is evident from the results that the new fingerprint is almost unaffected by JPEG compression attack on the images. This is not surprising, because the new device-specific fingerprint resides in the low- and mid-frequency band, so even if JPEG compression removes the high-frequency components in the image, the proposed method still effectively captures the non-PRNU device fingerprint and identify the source device of the images.

## 5. Conclusions

In this paper, we explore the existence of a new device-specific fingerprint other than the PRNU in the low- and mid-frequency bands of images for source-device identification. The presence of the new device fingerprint in the low- and mid-frequency bands of images is established by training a hybrid ResNet101–SVM system on downsampled and randomly sampled image patches. While existing data-driven approaches can learn features that can differentiate only camera brands or models, the experimental results suggest that the proposed system is effective in extracting the non-PRNU device-specific fingerprint and mapping the images to individual devices of the same model. The source device identification based on the new device-specific fingerprint presented in this paper provides significantly more accurate and reliable results compared to earlier data-driven approaches that depend on the PRNU fingerprint. Moreover, PRNU-based approaches demand proper spatial alignment of the images to identify the source camera. Nonetheless, the location-independent, global, and stochastic characteristics of our new device fingerprint that learned from randomly sampled patches make it possible to identify the source camera even with spatial misalignment of the images. We also investigate the reliability of the new fingerprint from images subjected to gamma correction, rotation, and JPEG compression. We show that the new fingerprint is effective and robust in identifying the source camera of images subjected to aggressive JPEG compression. With these promising results, we establish the presence of a robust device-specific fingerprint that can be used for image forensics applications.

In the proposed work, we bring to light the existence of and identified the characteristics of a novel device-specific fingerprint in images. There are several sources of imperfections that imprint traces on images at various stages of the image acquisition process. We note that a much more extensive investigation is necessary to determine the hardware component and/or the in-camera processing procedures that embed the new fingerprint in images. Nevertheless, this initial outcome is promising. Therefore, in-depth analysis of the origin of the new device-specific fingerprint is a topic of further interest for future research.

## Figures and Tables

**Table 1:** Devices in different datasets used in our experiments.

Dataset	Model	No. of Devices	No. of Images from Each Device	Resolution
**Vision**	iPhone 5c	2	204	3264 × 2448
	iPhone 4s	2	200	3264 × 2448
**Warwick**	Fujifilm X-A10	2	197	3264 × 2448
**Daxing**	Huawei P20	2	190	2976 × 3968
	iPhone 6	3	190	3264 × 2448
	iPhone 7	3	190	4032 × 3024
	iPhone 8 Plus	1	190	4032 × 3024
	Oppo R9	2	190	3120 × 4160
	Xiaomi 4A	5	190	3120 × 4160
**UNISA2020**	Nikon D90	10	100	4288 × 2848
**Custom-built**	Redmi Note 3	2	200	4608 × 3456
	Asus Zenfone MaxProM1	2	250	4608 × 3456

**Table 2:** PCE evaluation of the PRNUs extracted from the original and downsampled images with respect to ${\hat{F}}_{nat}$ and ${\hat{F}}_{flat}$.

Device	Average PCE of PRNUs of 30 Original Images with Respect to ${\hat{F}}_{\mathrm{nat}}$	Average PCE of PRNUs of 30 PRNU-Free Images with Respect to ${\hat{F}}_{\mathrm{nat}}$	Average PCE of PRNUs of 30 PRNU-Free Images with Respect to ${\hat{F}}_{\mathrm{flat}}$
**iPhone 5c-1**	16,067	1.52	2.19
**iPhone 5c-2**	9848	1.96	2.93
**iPhone 4s-1**	17,674	1.22	1.89
**Fujifilm X-A10-1**	1095	1.51	-
**Redmi Note 3-1**	14,845	1.51	-

**Table 3:** Source-camera identification performance based on the non-PRNU fingerprint extracted from downsampled and randomly sampled images.

Exp.	Device Used	Testing Accuracy (%)		
		Downsampling from Original Images	Random Sampling from Original Images	Random Sampling from Downsampled Images
**1**	2 iPhone 5c	97.06	83.86	84.44
**2**	2 iPhone 4s	98.00	85.70	86.38
**3**	2 Fujifilm X-A10	95.92	84.39	86.27
**4**	2 Redmi Note 3	97.00	85.38	92.26
**5**	2 Asus Zenfone Max Pro M1	95.16	84.25	86.24
**6**	5 Xiaomi 4A	99.57	94.01	95.79
**7**	16 devices of 6 different models from the Daxing dataset	99.20	98.15	99.58

**Table 4:** Performance of the new device-specific fingerprint in the open-set scenario.

Cameras Used to Train the Fingerprint Extractor	Testing Accuracy Achieved on Open-Set Cameras (%)				
	6 Oppo R9 (6 Cameras)	2 Asus Zenfone Max Pro M1 2 iPhone 5 4 Vivo X9 (8 Cameras)	2 Fujifilm X-A10 2 Redmi Note 5 Pro 6 Oppo R9 (10 Cameras)	2 Fujifilm X-A10 2 Redmi Note 5 Pro 2 Redmi Note 3 6 Oppo R9 (12 Cameras)	33 Devices from 11 Brands of VISION Dataset
**2 iPhone 5c**	99.40	92.16	90.40	83.33	63.78
**5 Xiaomi 4A**	99.70	96.61	91.06	86.52	65.37

**Table 5:** Performance comparison of proposed methods with state-of-the-art techniques.

Method	Testing Accuracy (%)			
	2 iPhone 4s	2 Fujifilm X-A10	2 Redmi Note 3	16 Devices (Daxing Dataset)
**CNN + SVM [34]**	50.00	50.00	50.00	6.25
**CNN-based multi-classifier [36]**	76.00	58.16	75.00	76.60
**CNN with 6 layers [37]**	73.00	45.00	76.00	21.15
**CNN with 11 layers [38]**	50.00	50.00	50.00	6.25
**LBP + modified AlexNet [39]**	64.71	50.00	53.00	6.25
**Residual neural network [43]**	80.00	81.63	79.00	91.88
**Content-adaptive fusion network [44]**	70.00	59.00	62.73	51.06
**Proposed downsampling from original images**	**98.00**	**95.92**	**97.00**	**99.20**
**Proposed random sampling from original images**	**85.70**	**84.39**	**85.38**	**98.15**
**Proposed random sampling from downsampled images**	**86.38**	**86.27**	**92.26**	**99.58**

**Table 6:** Performance comparison of proposed methods with state-of-the-art techniques on UNISA2020 dataset.

Method	Testing Accuracy (%)
**CNN + SVM [34]**	25.00
**CNN-based multi-classifier [36]**	74.05
**CNN with 6 layers [37]**	75.53
**CNN with 11 layers [38]**	62.27
**Residual neural network [43]**	77.12
**Content-adaptive fusion network [44]**	38.14
**Proposed random sampling from original images**	**96.02**

**Table 7:** Identification accuracy (%) of the proposed method under various image manipulations.

Device Used for the Experiment	Original	Gamma Correction		Rotation			JPEG Compression		
		γ=0.7	γ=1.4	$\theta =15^{\circ }$	$\theta =30^{\circ }$	$\theta =90^{\circ }$	Q=90%	Q=50%	Q=20%
**2 iPhone 5c**	97.06	97.06	96.08	96.08	87.25	91.18	97.06	96.08	96.08
**2 Fujifilm X-A10**	95.92	95.92	94.95	91.84	88.78	85.71	95.92	95.92	95.92
**2 Redmi Note 3**	97.00	97.00	94.00	97.00	94.00	83.00	97.00	97.00	96.00

## Data Availability

Publicly available datasets were analyzed in this study. This data can be found here: https://lesc.dinfo.unifi.it/VISION/; https://github.com/xyhcn/Daxing; https://github.com/DIFLabUnisa/CHIDataset.
